# Topical roflumilast 0.3% cream for mild hidradenitis suppurativa: A prospective case series

**DOI:** 10.1016/j.jdcr.2025.12.016

**Published:** 2025-12-17

**Authors:** Nagasai C. Adusumilli, Nikkia Zarabian, Mina Farah, Emily C. Murphy, Adam J. Friedman

**Affiliations:** Department of Dermatology, The George Washington University School of Medicine and Health Sciences, Washington, District of Columbia

**Keywords:** hidradenitis suppurativa, immunodermatology, immunomodulation, medical dermatology, pruritus, roflumilast

## Introduction

The United States Food and Drug Administration-approved biologics have expanded treatment options for moderate to severe hidradenitis suppurativa (HS). With newly approved medications and increased awareness by advocacy organizations, more patients are seeking care and presenting earlier with milder disease. Currently, no targeted therapies exist for mild HS, which is often managed with repeated cycles of topical and intralesional corticosteroids, topical and systemic antibiotics, and systemic androgen antagonists.^1^^,^^2^ However, overuse of corticosteroids in intertriginous regions of the body and antibiotic resistance are concerns of the current management paradigm for mild HS.^3^ Newer topical agents approved for other indications, such as ruxolitinib and clascoterone, have shown off-label potential for HS.^4^, ^5^, ^6^ Off-label oral roflumilast has been reported for severe disease burden refractory to biologics, suggesting a role for phosphodiesterase 4 inhibition in HS.^7^ We postulated that once-daily off-label topical roflumilast 0.3% cream, approved for plaque psoriasis, could improve mild HS.

## Case series

In our prospective case series, 3 patients with Hurley stage 1 disease were assessed at baseline, Day 30, and Day 60. Inclusion and exclusion criteria resembled those of the topical ruxolitinib exploratory trial (NCT04414514). Patients on stable dosing of biologic or antiandrogen therapy (≥6 months), and off medicated topicals or corticosteroids 4 weeks before and throughout the study, were included. Chlorhexidine wash and systemic baseline medications, excluding antibiotics and corticosteroids, were permitted and continued at the same dosing throughout the study. Chlorhexidine wash was standardized to every other day. Topical roflumilast 0.3% cream, using samples, was initiated once daily to affected sites and continued even without active flares. At the 3 evaluations, the absolute nodule/abscess counts and patient-reported intensity of pain, itch, odor, and drainage over the preceding week from the HS Symptom Questionnaire, were recorded.^8^

As discussed in Table I, Patients A and B were both 36-year-old females with Hurley Stage 1 HS who were treated with concurrent chlorhexidine wash every other day. Patient C was a 31-year-old female with Hurley Stage 1 HS who additionally started spironolactone 100 mg daily due to greater nodule burden at baseline. Clinical effects of spironolactone are typically appreciated at 3 months,^9^ likely avoiding confounding within the 60-day study period. All patients were treatment-naïve at the baseline visit or met the inclusion criteria as above.

**Table I:** Concurrent treatments and outcomes

Patient	Concurrent treatments	Day 0		Day 30		Day 60	
		# of nodules and abscesses	Hidradenitis suppurativa symptom questionnaire scores	# of nodules and abscesses	Hidradenitis suppurativa symptom questionnaire scores	# of nodules and abscesses	Hidradenitis suppurativa symptom questionnaire scores
A 36 yo F Stage 1	Chlorhexidine every other day (QOD)	2	Pain: 0	0	Pain: 0	0	Pain: 0
			Itch: 3		Itch: 0		Itch: 0
			Odor: 0		Odor: 0		Odor: 0
			Drainage: 0		Drainage: 0		Drainage: 0
B 36 yo F Stage 1	Chlorhexidine QOD	2	Pain: 2	1	Pain: 2	0	Pain: 0
			Itch: 4		Itch: 0		Itch: 0
			Odor: 2		Odor: 3		Odor: 0
			Drainage: 2		Drainage: 1		Drainage: 0
C 31 yo F Stage 1	Spironolactone 100 mg daily Chlorhexidine QOD	6	Pain: 3	0	Pain: 0	0	Pain: 0
			Itch: 6		Itch: 0		Itch: 0
			Odor: 2		Odor: 0		Odor: 0
			Drainage: 6		Drainage: 0		Drainage: 0

**Fig 1:**
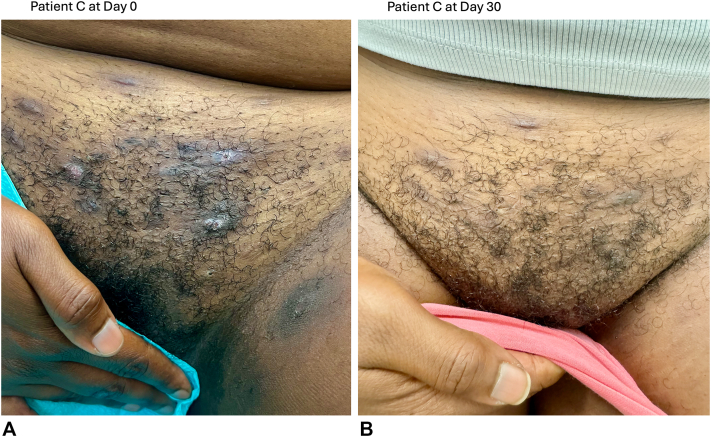
Representative images of clinical improvement of hidradenitis suppurativa. **Panel A,** At the baseline evaluation, Patient C had 6 actively inflamed nodules on the mons pubis and upper medial thighs. **Panel B,** At the 1-month reevaluation, Patient C continued to have dyspigmentation and thin, superficial scarring, without any active, inflamed nodules or abscesses.

After 30 and 60 days, all patients reported fewer nodules with no new lesions (Table I). Patients A and C experienced complete resolution of nodule count and cardinal symptoms by 1 month, with the clearance maintained up to the 2-month re-evaluation. Although 1 painful nodule persisted for Patient B after 1 month, she did experience nodule and symptom clearance by 2 months. Patient C had the highest initial burden of objective nodules and subjective symptoms, but her clinical improvement throughout the 2 months resembled that of Patients A and B (Fig 1). Notably, all 3 patients, including Patient C with an initially high HS Symptom Questionnaire itch score, reported alleviation of itch by 1 month and continued itch relief through 2 months. Patients were monitored for tolerability and adverse events, including burning, stinging, and gastrointestinal distress. Patients reported no side effects and adverse events, even when applying topical roflumilast 0.3% cream to multiple involved skin folds.

## Discussion

Topical roflumilast 0.3% cream may represent a promising therapy for patients with mild HS. This potential likely stems from its mechanism of tightly controlling the amplitude and duration of cyclic adenosine monophosphate’s (cAMP) effects. Intracellular cAMP downregulates proinflammatory cytokines of the Th1 and Th17 pathways via inhibition of the protein kinase A-nuclear factor kappa B (PKA-NFkB) cell signaling.^10^ PDE4 degrades cAMP to AMP; inhibition of PDE4 by roflumilast increases intracellular cAMP levels, which in turn suppresses chemotaxis, reduces the release of cytotoxic mediators, and limits inflammatory cell infiltration.^11^ Elevated cAMP also exerts immunomodulatory and anti-inflammatory effects, leading to downregulation of proinflammatory cytokines such as tumor necrosis factor alpha, interleukin (IL)-17, and other cytokines implicated in the pathophysiology of HS including IL-1, IL-23, and IL-36.^10^^,^^11^ In this prospective case series, we demonstrate reduction of physician-observed nodule count and patient-reported symptoms by 1 month, along with sustained improvement at 2 months. The small sample size of 3 patients, absence of a control group, few baseline lesions in the context of a spontaneously remitting disease when at early stages, and variable concomitant regimens limit this study. However, our results suggest an anti-inflammatory benefit of topical roflumilast in mild HS, and its potential as an adjuvant in moderate cases. Larger, randomized, controlled studies are further warranted.

## Conflicts of interest

AF is a speaker and a consultant for Arcrutis.
